# Cross‐Peaks in Simple Two‐Dimensional NMR Experiments from Chemical Exchange of Transverse Magnetisation†

**DOI:** 10.1002/anie.201903245

**Published:** 2019-05-24

**Authors:** Christopher A. Waudby, Tom Frenkiel, John Christodoulou

**Affiliations:** ^1^ Institute of Structural and Molecular Biology University College London Gower Street London WC1E 6BT UK; ^2^ MRC Biomedical NMR Centre Francis Crick Institute Midland Road London NW1 1AT UK

**Keywords:** chemical exchange, kinetics, lineshape analysis, NMR spectroscopy

## Abstract

Two‐dimensional correlation measurements such as COSY, NOESY, HMQC, and HSQC experiments are central to small‐molecule and biomolecular NMR spectroscopy, and commonly form the basis of more complex experiments designed to study chemical exchange occurring during additional mixing periods. However, exchange occurring during chemical shift evolution periods can also influence the appearance of such spectra. While this is often exploited through one‐dimensional lineshape analysis (“dynamic NMR”), the analysis of exchange across multiple chemical shift evolution periods has received less attention. Here we report that chemical exchange‐induced cross‐peaks can arise in even the simplest two‐dimensional NMR experiments. These cross‐peaks can have highly distorted phases that contain rich information about the underlying exchange process. The quantitative analysis of such peaks, from a single 2D spectrum, can provide a highly accurate characterisation of underlying exchange processes.

NMR spectroscopy is an exceptionally powerful technique for the label‐free analysis of intramolecular dynamics and chemical exchange, with a range of applications to fluxional molecules,^1^ supramolecular chemistry and host/guest interactions,^2^ and biomolecular function and interactions.^3^ By using radiofrequency pulses to perturb the magnetisation of systems in dynamic equilibrium, the associated chemical exchange processes can be characterised with high precision across a wide range of timescales. A variety of experiments have been developed towards this end, including NOESY (also referred to in this context as EXSY),^4^ ZZ‐exchange spectroscopy,^5^ chemical exchange saturation transfer (CEST),^6^ and CPMG/*R*_1ρ_ relaxation dispersion.^3^

All of the above experiments are based on the characterisation of exchange occurring during a specific mixing time within the pulse sequence. However, resonance lineshapes are also directly sensitive to chemical exchange processes, provided that the exchange rate, *k*_ex_, is within one or two orders of magnitude of the frequency difference, Δ*ω*, between the exchanging resonances. Therefore, in a long‐standing approach termed lineshape analysis or “dynamic NMR”, one‐dimensional (1D) spectra may be fitted in a least‐squares sense to solutions of the Bloch–McConnell or Liouville–von Neumann equations^7^, ^8^ that govern the evolution of magnetisation, in order to characterise the chemical exchange process. The approach has also been extended to two‐dimensional (2D) lineshape analysis: the fitting of 2D NMR spectra, by direct simulation of the relevant pulse sequence.^9^ This approach, and the associated TITAN analysis software, has since found applications to a variety of biomolecular interactions.^10^, ^11^

As part of an effort to validate the accuracy of 2D lineshape analysis, we conducted a series of measurements of the small molecule *N*,*N*‐dimethyl‐trichloroacetamide (DMTCA) (Figure 1 A,B), the two methyl groups of which undergo exchange by rotation about the amide bond with a rate of 125 s^−1^ at 298 K.^12^, ^13^, ^14^ DMTCA is a simple molecule, with no resolved homonuclear scalar couplings, but serves to illustrate fundamental principles that will be equally applicable to more complex molecules and exchange processes.

**Figure 1:**
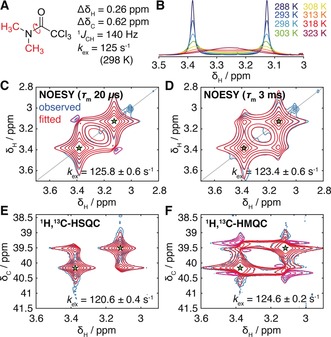
A) Chemical structure and key spectral parameters of *N*,*N*‐dimethyltrichloroacetamide (DMTCA). B) 1D ^1^H NMR spectra of DMTCA acquired at 16.44 T as a function of temperature as indicated. C–F) Observed (blue/cyan) and fitted (red/magenta) NOESY, HSQC, and HMQC spectra of DMTCA (298 K, 16.44 T). Positive contours are shown in blue/red and negative contours in cyan/magenta. Asterisks indicate fitted resonance positions.

We first performed a series of 2D ^1^H,^1^H NOESY (aka EXSY) experiments to characterise chemical exchange within DMTCA (Figure 1 C,D). An essentially complete mathematical description of the NOESY experiment for a one‐spin system in the presence of exchange has been given by Jeener et al.,^4^ describing the observed magnetisation ***M*** ^+^ as a function of the evolution periods *t*_1_ and *t*_2_, and a mixing time, *τ*_m_ [Eq. (1)].

(1) M+t1,τm,t2=e(iΩ-R+K)t2e(-L+K)τmRee(iΩ-R+K)t1M0

Here **K**, **Ω**, **R**, and **L** are the superoperators describing chemical exchange, chemical shift evolution, transverse relaxation, and longitudinal relaxation, respectively. The observed magnetisation ***M*** ^+^ is cosine modulated in *t*_1_; sine modulation can be obtained by taking the imaginary component in Equation (1). The NOESY pulse sequence is designed to select *z* magnetisation that is present in the mixing time *τ*_m_, and we will consider short mixing times such that longitudinal relaxation (or cross‐relaxation) can be neglected. In conventional applications of the experiment, in‐phase cross‐peaks are generated as a result of chemical exchange of *z* magnetisation during the mixing time, and the experiment thus creates a readily interpreted “map” of the propagator $e^{K\tau_{m}}$ for the exchange superoperator.

For very short mixing times, the propagator $e^{K\tau_{m}}$ reduces to the identity operator, and no exchange cross‐peaks are expected. However, when such an experiment was acquired for DMTCA (with a near‐zero 20 μs mixing time), cross‐peaks were unexpectedly observed at frequencies (*ω*_A_, *ω*_B_) and (*ω*_B_, *ω*_A_), with intensities ca. 5 % that of the diagonal peaks (Figure 1 C). In contrast to the diagonal peaks, the cross‐peaks were not absorption mode, but had a partially dispersive lineshape. When a non‐zero mixing time was used, stronger cross‐peaks were observed, as expected from the exchange of *z* magnetisation, although a partially dispersive character could again be discerned (3 ms, Figure 1 D). We note that zero‐quantum artifacts cannot account for these cross‐peaks, as zero‐quantum coherences formed during *τ*_m_ give rise to antiphase dispersive signals that will be strongly suppressed because the exchange‐broadened linewidth (ca. 20 Hz) is much larger than the ^4^ *J*_HH_ coupling constant (estimated to be ca. 0.4 Hz by analogy with a related molecule^15^).

The origin of these cross‐peaks can be understood through analogy with non‐equilibrium stopped‐flow NMR.^16^, ^17^ Following Christianson and Landis,^17^ we consider first only “A” spins present at the beginning of the *t*_1_ evolution period, and work in the rotating frame of spin B. As the A spins precess, those that chemically exchange to state B have zero frequency in this frame and do not precess further (barring further chemical exchange). In other words, the A magnetisation vector can be envisaged as “dropping” B spins behind it as it precesses (Figure 2 A, Movies S1 and S2 in the Supporting Information). As illustrated by this schematic, the total magnetisation of these B spins, *M*_B_, precesses with offset Ω_A_=*ω*_A_−*ω*_B_ about a point displaced from the origin. This can be represented (Figure 2 B) as the vector sum of magnetisation, *M*′_B_, along +*y*, and magnetisation, *M′*′_B_, that is initially along −*y* and precesses with offset Ω_A_. Considered in the laboratory frame, evolution of *M*_B_ therefore gives rise to two dispersive signals, of opposite phases, at frequencies ω_A_ and ω_B_ (Figure 2 C). By symmetry, initial B spins exchanging to A during *t*_1_ give rise to identical dispersive signals. In 1D spectra, these signals are not resolved but contribute to the frequency shifts that occur in slow/intermediate exchange, leading to coalescence. However, the dispersive signals can be resolved directly using 2D NMR techniques, as further evolution during *t*_2_ reveals the origin of the signals in *t*_1_ (Figure 2 C). Therefore, exchange‐induced cross‐peaks appear with frequencies (*ω*_A_, *ω*_B_) and (*ω*_B_, *ω*_A_), as demonstrated experimentally above (Figure 1 C). These cross‐peaks will be observable provided *R*_2,0_≲*k*_ex_≲Δ*ω*, that is, when exchange is comparable to or faster than the decay of transverse magnetisation in the absence of exchange broadening (*R*_2,0_), and up to the coalescence point, beyond which only a single resonance is observed.

**Figure 2:**
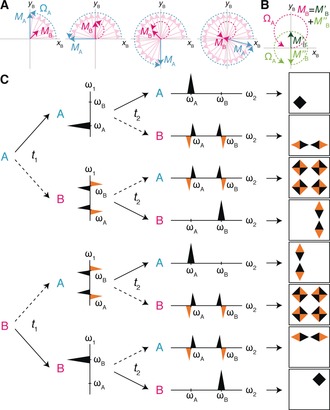
Origin of cross‐peaks due to chemical exchange during chemical shift evolution periods. A) Evolution of an initial population of A spins (blue), undergoing exchange to spin B, depicted in the rotating frame of spin B. Individual B spins are shown in pink, and the net magnetisation vector in magenta. Trajectories followed by magnetisation vectors are shown with dashed lines. A more detailed analysis of the trajectory of spin B magnetisation is shown in (B). Adapted from Christianson and Landis.^17^ C) Components of 2D lineshapes in *ω*_1_ and *ω*_2_ frequency domains arising from chemical exchange between spins A and B during consecutive chemical shift evolution periods, *t*_1_ and *t*_2_. Solid arrows show the pathway followed by spins that do not undergo exchange in the indicated time period, while dashed arrows show the pathway of exchanging spins. The final column illustrates the resulting contribution from each pathway to absorption, dispersion, and double‐dispersion components of the observed 2D lineshape.

Having established a conceptual basis for these unexpected exchange cross‐peaks, we sought to fit the observed spectra quantitatively, in order to verify our understanding of the process and to characterise the kinetics of the underlying exchange process. 2D lineshape fitting was performed using TITAN^9^ [which in this case reduces to the numerical integration of Equation (1)], and fits of NOESY spectra with both zero and non‐zero mixing times reproduced the observed spectra very closely (Figure 1 C,D), with fitted exchange rates (indicated in the figures) consistent with published results.^12^, ^13^, ^14^ We note that while in principle cross‐peaks formed at zero mixing times are dispersive and integrate to zero, in practice the long dispersive tails render this impractical and lineshape fitting is therefore recommended.

We also investigated the occurrence of exchange cross‐peaks in other simple NMR experiments, and found that cross‐peaks were also formed in COSY experiments, with a relative intensity of ca. 10 % of the diagonal peaks (Figure S1A). The precise form of these cross‐peaks was different from those observed in the NOESY experiment (Figure 1 C), which reflects differences in the transfer of magnetisation between *t*_1_ and *t*_2_ evolution periods between the two pulse sequences, but again, high‐quality fits and measurement of the exchange rate could be obtained by 2D lineshape analysis (Figure S1A).

We next explored the occurrence of exchange‐induced cross‐peaks in heteronuclear single‐ and multiple‐quantum coherence experiments (HSQC and HMQC, respectively). Heteronuclear experiments are typically more complex pulse sequences, containing extended coherence transfer periods and *zz* filters, which create multiple possibilities for chemical exchange to affect the spectra. Chemical exchange broadening is well understood to reduce the efficiency of coherence transfer in DEPT and INEPT experiments near intermediate exchange regimes,^18^, ^19^ but the possibility of coherent exchange of transverse magnetisation between states (outside of the fast exchange regime) does not appear to have been recognised.

Coherent transverse magnetisation exchange may be predicted using a simple argument (illustrated in Figure 3 A for the 1/4 *J*−π(I_x_+S_x_)−1/4 *J* sequence that forms part of an INEPT transfer, and assuming no change in the scalar coupling constant between states). An initial population of A spins, considered in the rotating frame of spin A, will evolve with frequency ±π*J* depending on the state of the coupled heteronucleus. Spins that chemically exchange into state B during this period will receive an additional phase shift, resulting in the fanning out of their magnetisation vectors. By following these spins through the rest of the sequence, it may thus be observed that spin B magnetisation can be generated, with a phase shift, from the initial spin A magnetisation. The extent of this exchange‐mediated coherence transfer from state A to state B depends on the frequency difference between the states and the total evolution time, 2*τ*=1/2 *J*, relative to the chemical exchange rate, *k*_ex_. Exact numerical calculations (Figure 3 B,C) show that when both the scalar coupling and frequency difference are comparable to the exchange rate (center of diagrams), a nontrivial population of B magnetisation is generated with a phase shift dependent on the frequency difference, as predicted from our schematic argument above (Figure 3 A).

**Figure 3:**
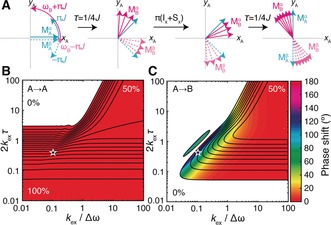
Analysis of chemical exchange during the 1/4 *J*−π(I_x_+S_x_)−1/4 *J* scalar coupling evolution period that forms part of an INEPT coherence transfer sequence. A) Evolution of an initial population of A magnetisation throughout the coherence transfer sequence, depicted in the rotating frame of spin A. A magnetisation is shown in blue, and B magnetisation is shown in magenta. The α/β spin state of the coupled heteronucleus is indicated with solid/dashed lines, respectively. B,C) Transfer efficiencies, calculated across the 1/4 *J*−π(I_x_+ S_x_)−1/4 *J* mixing sequence, for an initial population of A magnetisation in chemical exchange with state B. Contour lines are shown at 5 % intervals and shading indicates the acquired phase shift.

To explore the above analysis experimentally, natural‐abundance ^1^H,^13^C HSQC, and HMQC spectra of DMTCA were acquired at 298 K and 16.44 T (Figure 1 E,F). Under these conditions, 2 *k*_ex_ *τ*=0.45 and *k*_ex_/Δ*ω*_H_=0.11 (marked by an asterisk in Figure 3 B,C), which is a favourable regime to observe the predicted coherence transfer effects. Again, unexpected exchange‐induced cross‐peaks were observed in both experiments, with a particularly complex phase structure in the case of the HMQC experiment (Figure 1 F). This may be rationalised since in the HSQC experiment *zz* filters suppress phase distortions from the first INEPT transfer and some phase distortions arising from exchange during *t*_1_. In contrast, in the HMQC experiment all parts of the pulse sequence contribute to the phase of the observed magnetisation, resulting in remarkably complex lineshapes. HMQC spectra acquired at multiple fields to probe the effect of varying Δ*ω* clearly show that larger frequency differences are associated with larger phase distortions (Figure S2), as predicted from Figure 3 C, while applying XY16 CPMG pulse trains^20^ during HMQC coherence transfer periods suppresses the build‐up of phase shifts, greatly simplifying the structure of the exchange cross‐peaks (Figure S3). Again, high‐quality fits and measurement of the exchange rate could be obtained by 2D lineshape analysis (Figure S1A). We note that coherence transfers via the three‐bond scalar coupling ^3^ *J*_CH_ (not resolved, but estimated to be ca. 3.5 Hz by analogy with a related molecule^15^) will have ca. 0.01 % of the efficiency of the one‐bond transfer and cannot account for the amplitudes of the observed cross‐peaks.

Finally, to illustrate a potential application of these analyses, we examined the temperature dependence of exchange within DMTCA using a series of NOESY and HMQC experiments (Figure 4 and Figure S4). The HMQC experiment was selected, as this was established above to be the most sensitive experiment for observing phase distortions. Exchange rates were determined from 2D fitting of individual spectra, and varied from 19.0±0.6 s^−1^ at 278 K to 482±4 s^−1^ at 313 K. NOESY and HMQC results both fitted well to the Eyring equation (Figure 4), fully consistent with previous measurements (Δ*H* ^≠^=67.4±3.9 kJ mol^−1^ and Δ*S* ^≠^=15±13 kJ mol^−1^ K^−1^).^13^ We note that at higher temperatures, 2D lineshape analysis of the NOESY experiments, acquired with very short mixing times, was also required in order to characterise the exchange process (Figure S4).

**Figure 4:**
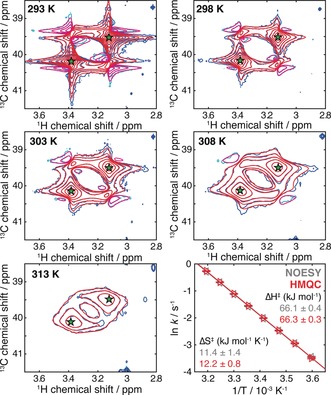
Temperature dependence of chemical exchange in DMTCA, measured by 2D lineshape analysis of HMQC spectra (coloring as in Figure 1). Measured rates were fitted to the Eyring equation as shown, together with measurements acquired using NOESY experiments (Figure S4).

In conclusion, chemical exchange is well known to induce changes in NMR chemical shifts and intensities, which have been exploited through, for example, longitudinal magnetisation exchange, lineshape analysis, and relaxation dispersion experiments.^3^ In this work, we have shown that chemical exchange may also give rise to detectable phase shifts and coherent transfers of transverse magnetisation. For suitable exchange regimes, this provides rapid and accurate characterisation of exchange in a single 2D spectrum. We have focussed our examples on the simple molecule DMTCA, which does not contain any large homonuclear scalar couplings, but the presence of scalar couplings (although not yet implemented within TITAN) is not expected to fundamentally alter this picture. The approach is also expected to be applicable to more complex multistep reactions. The analysis of transverse magnetisation exchange is free from complicating effects of cross relaxation, and may be a particularly useful complement to NOESY measurements where the exchange rate approaches the slow/intermediate exchange regime. More generally, our work highlights the complexity of spectral features that can arise from exchange and points to the importance of quantitative analysis in terms of the fundamental spin dynamics.

## Experimental Section

*N*,*N*‐dimethyltrichloroacetamide (DMTCA, CAS number 7291‐33‐0) was purchased from Fluorochem (UK), and diluted to 3 % (v/v) in CDCl_3_. NMR spectra were acquired on a Bruker Avance III NMR spectrometer operating at 16.44 T (corresponding to a ^1^H Larmor frequency of 700 MHz) and equipped with a QCI cryoprobe. Where indicated, additional experiments were performed using a Bruker Avance III NMR spectrometer operating at 11.74 T (500 MHz ^1^H Larmor frequency) and equipped with a Prodigy TCI cryoprobe, and a Bruker Avance III NMR spectrometer operating at 22.32 T (950 MHz ^1^H Larmor frequency) and equipped with a TCI cryoprobe. Unless otherwise indicated, spectra were acquired at 298 K. Acqusition and processing parameters are collated in Table S1.

Simulations of exchange during 1/4 *J*−π(I_x_+S_x_)−1/4 *J* and XY16‐CPMG scalar coupling evolution sequences were performed in MATLAB (R2016b, The MathWorks, Inc.), by numerical propagation of density operators in Liouville space, as previously described.^9^ Movies S1 and S2 in the Supporting Information were generated in Mathematica 11 (Wolfram Research Inc., Champaign, Illinois), by stochastic simulation of the evolution of 20 000 spins freely precessing in the *xy* plane, in the absence of relaxation. Two‐dimensional lineshape analysis was performed in TITAN (v1.6) and uncertainties in the fitted exchange rates were determined by bootstrap resampling.^9^

## Conflict of interest

The authors declare no conflict of interest.

## Supporting information

As a service to our authors and readers, this journal provides supporting information supplied by the authors. Such materials are peer reviewed and may be re‐organized for online delivery, but are not copy‐edited or typeset. Technical support issues arising from supporting information (other than missing files) should be addressed to the authors.

Supplementary

Supplementary

Supplementary
