# Supplementary material for: Cross‐Peaks in Simple Two‐Dimensional NMR Experiments from Chemical Exchange of Transverse Magnetisation
Source: Angew Chem Int Ed Engl. 2019 May 24;58(26):8784–8. doi: 10.1002/anie.201903245 (PMC6771647; doi:10.1002/anie.201903245)
Supplement: Supplementary file 1 — Supplementary [file ANIE-58-8784-s001.pdf]

Angewandte Chemie
Eine Zeitschrift der Gesellschaft Deutscher Chemiker

Supporting Information

Cross-Peaks in Simple Two-Dimensional NMR Experiments from Chemical Exchange of Transverse Magnetisation**

Christopher A. Waudby, Tom Frenkiel, and John Christodoulou*

anie_201903245_sm_miscellaneous_information.pdf
anie_201903245_sm_movie-S1.mov
anie_201903245_sm_movie-S2.mov

Table of Contents

Results and Discussion p. 2
Author Contributions p. 5

Results and Discussion

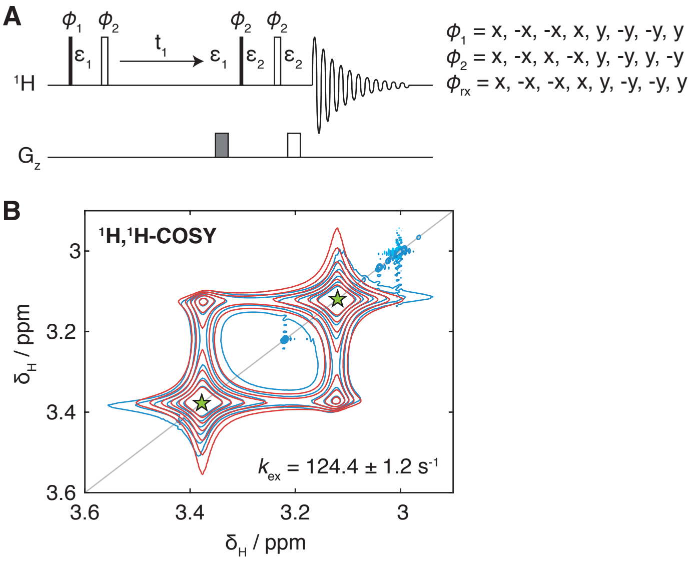

Figure S1. (A) gCOSY pulse sequence used in this work. ε_1 = ε_2 = 0.75 ms. Dark grey shading of gradient pulses indicates that the sign should be inverted for echo/anti-echo quadrature detection. (B) Observed (blue/cyan) and fitted (red/magenta) gCOSY spectra of DMTCA (298 K, 16.44 T). Positive contours are shown in blue/red and negative contours in cyan/magenta. Asterisks indicate fitted resonance positions.

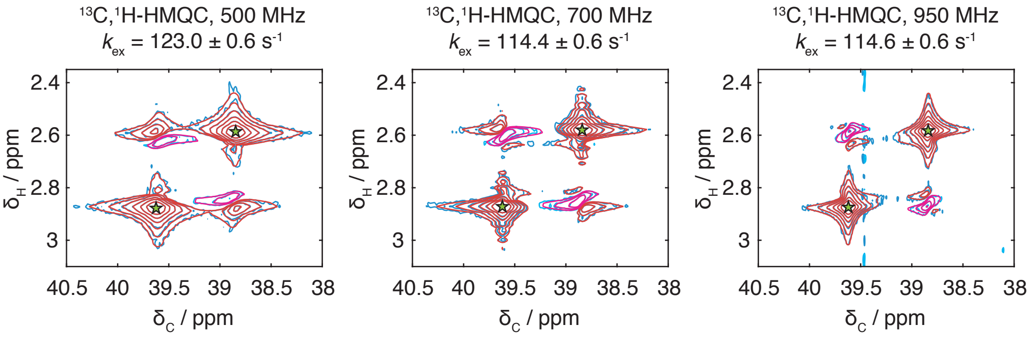

Figure S2. B_0 field dependence of ^{13}C,^{1}H-HMQC spectra of neat DMTCA, 298 K. Positive and negative contours are shown in blue and cyan respectively. Fitted spectra are shown with positive/negative contours in red/magneta respectively, with fitted exchange rates as indicated. Asterisks indicate fitted resonance positions.

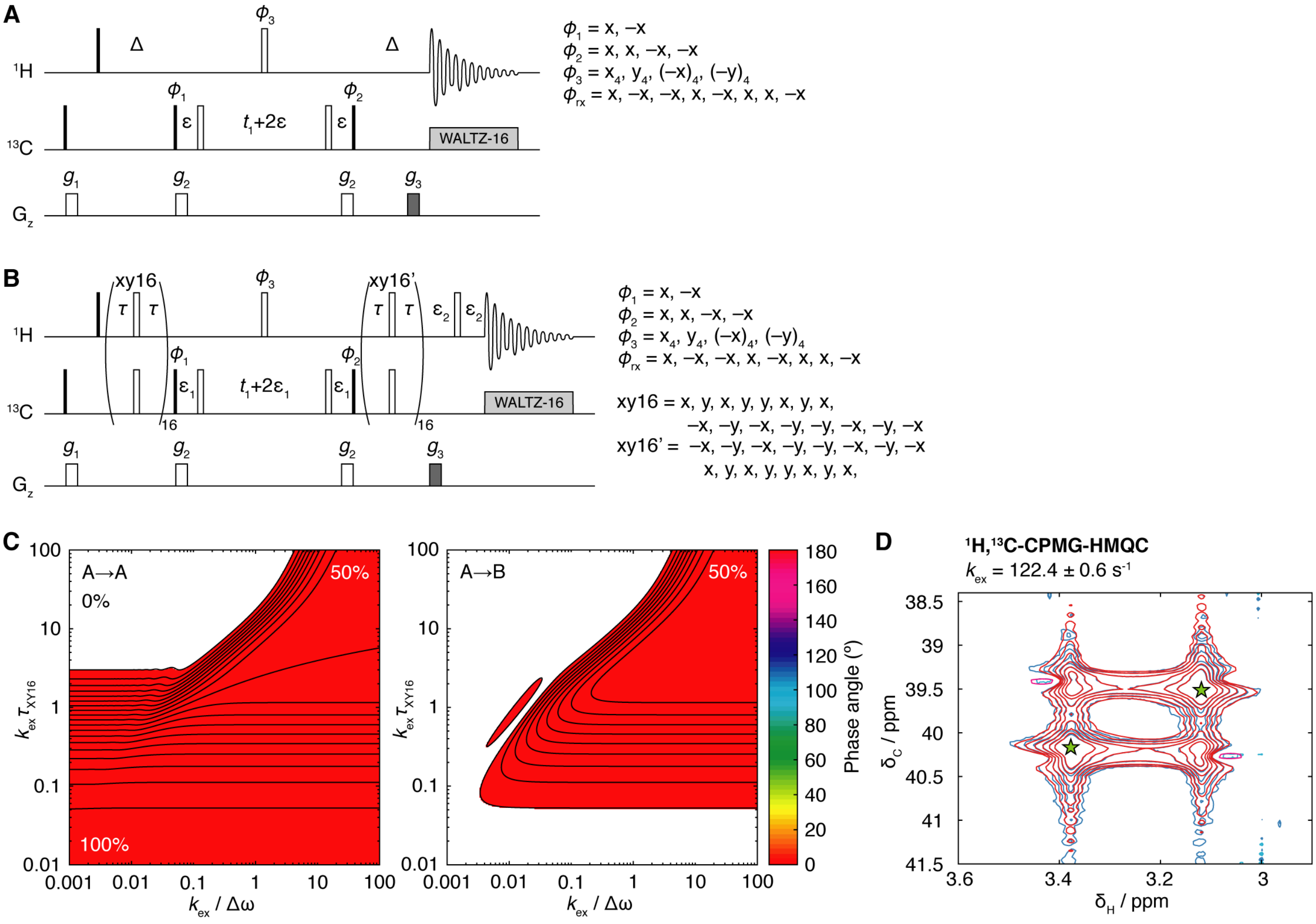

Figure S3. (A) gHMQC pulse sequence used in this work. ε = 0.75 ms, Δ = 1/2J = 3.57 ms, g_1 = 31% (1 ms), g_2 = 40% (0.6 ms), g_3 = 20.1% (0.6 ms). (B) CPMG-gHMQC pulse sequence used in this work. Bracketed pulses are applied according to the xy16 and time-reversed xy16' phase schemes as indicated. ε_1 = 0.75 ms, ε_2 = 0.75 ms, τ = 1/64J = 111.6 μs, g_1 = 31% (0.6 ms), g_2 = 40% (0.6 ms), g_3 = 20.1% (0.6 ms). Dark grey shading of gradient pulses indicates that the sign should be inverted for echo/anti-echo quadrature detection. (C) XY16 CPMG transfer efficiencies calculated for an initial population of A magnetization in chemical exchange with state B. Contour lines are shown at 5% intervals, and shading indicates the acquired phase shift. τ_{XY16}=1/2J is the total length of the XY16 coherence transfer period. (D) Observed (blue/cyan) and fitted (red/magenta) CPMG-gHMQC spectra of DMTCA (298 K, 16.44 T). Positive contours are shown in blue/red and negative contours in cyan/magenta. Asterisks indicate fitted resonance positions.

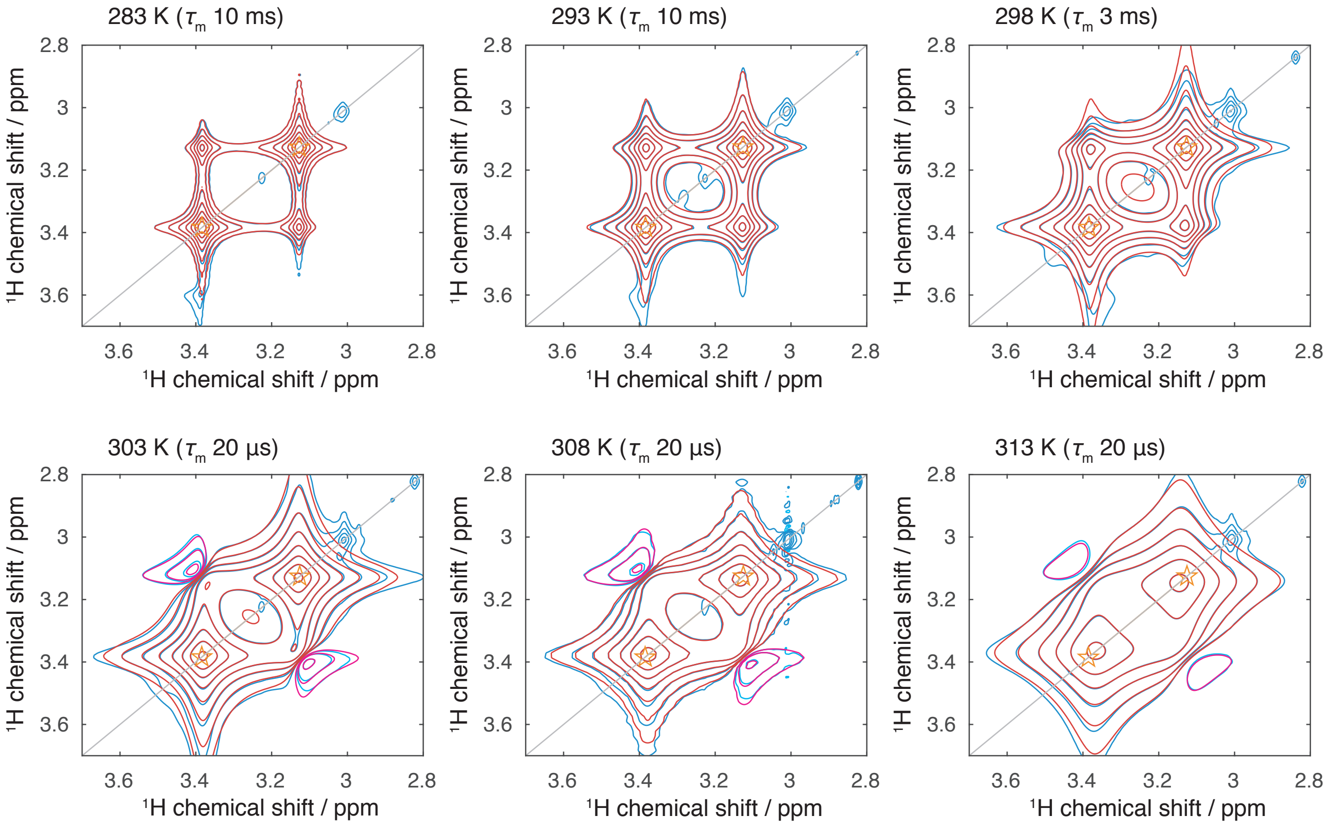

Figure S4. (A) Temperature dependence of chemical exchange in DMTCA, measured by 2D lineshape analysis of ^{1}H,^{1}H NOESY spectra. Observed (blue/cyan) and fitted (red/magenta) spectra; positive contours are shown in blue/red and negative contours in cyan/magenta. Asterisks indicate fitted resonance positions.

Movie S1. Stochastic simulation of the evolution of an initial population of 20000 ‘A’ state spins aligned along the *x* axis, in bidirectional exchange with state ‘B’ with rates $k_{AB} = k_{BA} = 0.05\ \Delta\omega_{AB}$, and plotted in the rotating frame of state B. Units of time are chosen such that $\Delta\omega_{AB} = 1$. Individual A and B spins are depicted with thin cyan and magenta arrows, respectively, while the total A and B magnetization is depicted with solid blue and red arrows respectively; the total B magnetization is magnified 5x. The evolution of the total A and B magnetization is traced out with solid blue and red lines respectively.

Movie S2. Stochastic simulation of the evolution of an initial population of 20000 ‘A’ state spins aligned along the *x* axis, in bidirectional exchange with state ‘B’ with rates $k_{AB} = k_{BA} = 0.05\ \Delta\omega_{AB}$, and plotted in a rotating frame mid-way between the frequencies of states A and B. Units of time are chosen such that $\Delta\omega_{AB} = 1$. Individual A and B spins are depicted with thin cyan and magenta arrows, respectively, while the total A and B magnetization is depicted with solid blue and red arrows respectively; the total B magnetization is magnified 5x. The evolution of the total A and B magnetization is traced out with solid blue and red lines respectively.

Table S1. Summary of acquisition and processing parameters.

Figure	Experiment	Field strength / MHz[a]	T / K	Direct dimension			Indirect dimension		
				Time domain (real points)	Acquisition time / ms	lb / Hz[b]	Time domain (real points)	Acquisition time / ms	lb / Hz[b]
1C	$^1H,^1H$ NOESY, 20 µs	700	298	16384	734	10	80	29	20
1D	$^1H,^1H$ NOESY, 3 ms	700	298	16384	734	10	80	29	20
1E	$^1H,^{13}C$ gHSQC	700	298	4096	184	1	160	100	3
1F	$^1H,^{13}C$ gHMQC	700	298	4096	184	1	160	100	3
4	$^1H,^{13}C$ gHMQC	700	293	4096	184	5	160	100	5
4	$^1H,^{13}C$ gHMQC	700	298	4096	184	5	160	100	5
4	$^1H,^{13}C$ gHMQC	700	303	4096	184	5	160	100	5
4	$^1H,^{13}C$ gHMQC	700	308	4096	184	10	160	100	10
4	$^1H,^{13}C$ gHMQC	700	313	4096	184	10	40	25	10
4	$^1H,^{13}C$ gHMQC	700	278	4096	184	5	160	100	10
4	$^1H,^{13}C$ gHMQC	700	283	4096	184	5	160	100	10
4	$^1H,^{13}C$ gHMQC	700	288	4096	184	5	160	100	5
S1	$^1H,^1H$ gCOSY	700	298	4096	184	1	384	91	1
S2	$^{13}C,^1H$ HMQC	500	298	1024	204	1	512	64	1
S2	$^{13}C,^1H$ HMQC	700	298	1024	145	1	200	48	1
S2	$^{13}C,^1H$ HMQC	950	298	2048	214	1	160	28	10
S3	$^1H,^{13}C$ CPMG-gHMQC	700	298	4096	184	1	80	50	1
S4	$^1H,^1H$ NOESY, 10 ms	700	278	16384	734	10	80	29	20
S4	$^1H,^1H$ NOESY, 10 ms	700	283	16384	734	10	80	29	20
S4	$^1H,^1H$ NOESY, 10 ms	700	288	16384	734	10	80	29	20
S4	$^1H,^1H$ NOESY, 10 ms	700	293	16384	734	10	80	29	20
S4	$^1H,^1H$ NOESY, 20 µs	700	303	16384	734	10	80	29	20
S4	$^1H,^1H$ NOESY, 20 µs	700	308	16384	734	5	80	29	5
S4	$^1H,^1H$ NOESY, 20 µs	700	313	16384	734	10	80	29	20

[a] Field strength is specified as the ^1H Larmor frequency. [b] Exponential line broadening.

Author Contributions

CW and TF conceived and planned the experiments. CW carried out the experiments, simulations and analysed the data with assistance from TF. JC supervised the project. CW wrote the manuscript with input from all authors.
